# Supplementary material for: Preliminary Findings on CTG Expansion Determination in Different Tissues from Patients with Myotonic Dystrophy Type 1
Source: Genes (Basel). 2020 Nov 7;11(11):1321. doi: 10.3390/genes11111321 (PMC7695006; doi:10.3390/genes11111321)

genes

MDPI

Figure S1. Representative SP-PCR gel with the different CTG size measurements. Analysis of the CTG repeat number estimates of patient P6 in blood, muscle and skin. Progenitor allele size (yellow), an estimate of the repeat length in the affected egg or sperm cell, is measured considering the shortest CTG size that is more represented in the four replicates. The estimated mode allele size (white) is measured based on the most abundant bands present in the four replicates. The longest CTG size (red) is calculated considering the highest intense signal present in the sample. M = molecular weight ladder; WT = wild type.

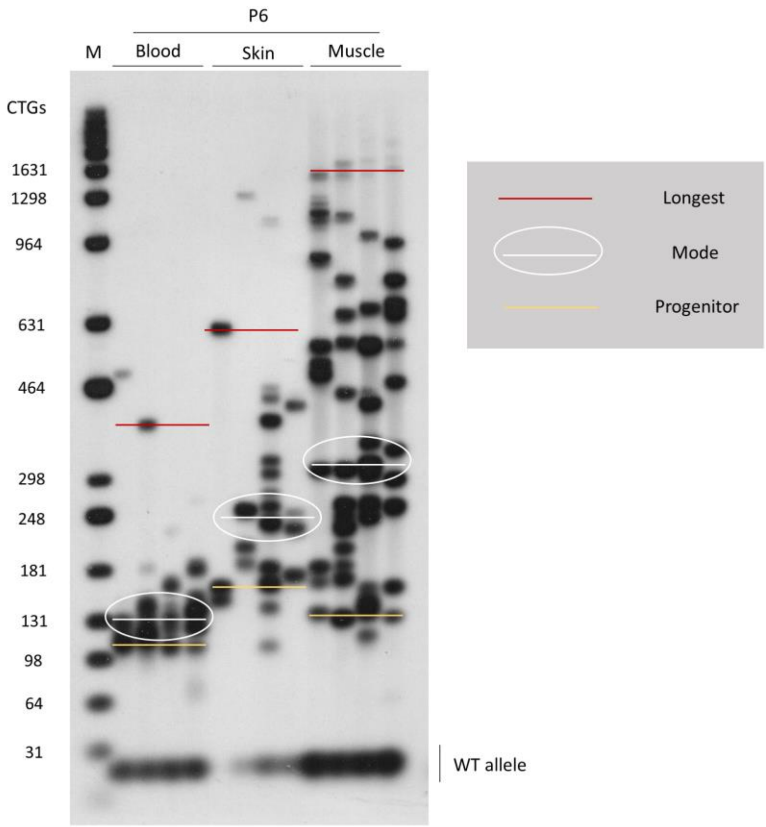

Figure S2. CTG size allele distribution in blood of the patient carrying interruptions vs. one patient carrying pure repeats. M = molecular weight ladder.

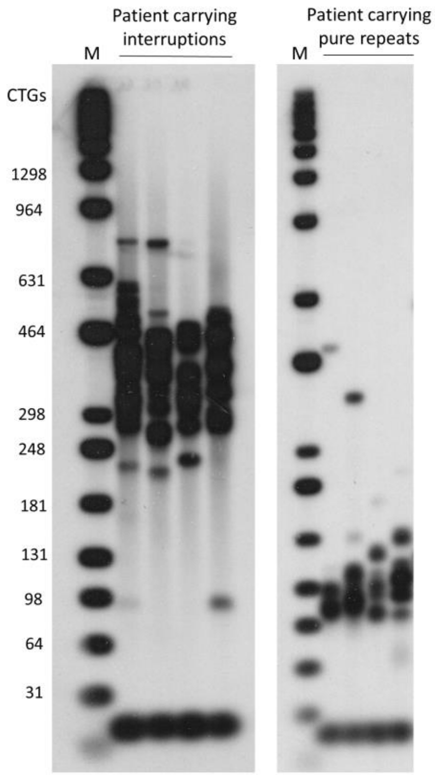

Supplement: Supplementary file 1 [file genes-11-01321-s001.pdf]
